# Culture-Confirmed Bacterial Sepsis and Invasive Fungal Infection in Preterm Infants: NICU Resource Burden, Major Morbidity, and Caregiver Psychological Distress

**DOI:** 10.3390/diseases14040120

**Published:** 2026-03-27

**Authors:** Sergiu Costescu, Adrian Ratiu, Bogdan Cerbu, Oana Cristina Costescu, Cosmin Citu, Aniko Maria Manea, Zoran Laurentiu Popa

**Affiliations:** 1 Doctoral School, “Victor Babes” University of Medicine and Pharmacy, Eftimie Murgu Square 2, 300041 Timisoara, Romania; costescu.sergiu@umft.ro; 2 Department of Obstetrics and Gynecology, “Victor Babes” University of Medicine and Pharmacy, Eftimie Murgu Square 2, 300041 Timisoara, Romania; ratiu.adrian@umft.ro (A.R.); citu.ioan@umft.ro (C.C.); popa.zoran@umft.ro (Z.L.P.); 3 Discipline of Neonatology, Faculty of Medicine, “Victor Babes” University of Medicine and Pharmacy, Eftimie Murgu Square 2, 300041 Timisoara, Romania; manea.aniko@umft.ro

**Keywords:** infant, premature, sepsis, candidemia, intensive care units, neonatal, caregivers

## Abstract

Background and Objectives: Very preterm infants are vulnerable to late-onset infection and prolonged NICU exposure, with potential downstream effects on caregiver health. We evaluated neonatal outcomes and caregiver psychosocial status across culture-confirmed infection phenotypes. Methods: We investigated a single-center prospective cohort (March 2023–December 2025) of 87 preterm infants assigned to one of three groups: no proven infection (*n* = 44), bacterial sepsis (*n* = 31), or candidemia (*n* = 12). Neonatal outcomes included a composite adverse endpoint (death or major morbidity) and resource utilization. Caregivers completed the SF-36, WHOQOL-BREF, HADS, PHQ-9, GAD-7, and Body Image Scale near discharge. Results: Candidemia occurred later than bacterial sepsis (day of life 17.8 ± 4.8 vs. 10.1 ± 3.9; *p* < 0.001) and had a longer time to effective therapy (23.3 ± 9.5 vs. 13.3 ± 5.3 h; *p* = 0.004). The composite adverse outcome was 27.3% in the no-infection group versus 54.8% in the bacterial group and 58.3% in the candidemia group (*p* = 0.025); ROP requiring treatment increased from 4.5% to 29.0% and 25.0% (*p* = 0.012). Length of stay rose from 39.7 ± 10.2 to 50.1 ± 11.9 and 60.9 ± 13.1 days (*p* < 0.001), and ventilation days from 15.7 ± 7.6 to 23.3 ± 7.5 and 34.2 ± 10.4 (*p* < 0.001). Caregiver SF-36 mental health (MCS) scores decreased from 44.7 ± 7.5 to 38.5 ± 6.0 and 36.7 ± 6.4 (*p* < 0.001), while PHQ-9 scores increased from 9.4 ± 3.9 to 11.6 ± 3.3 and 15.5 ± 4.6 (*p* < 0.001); NICU burden correlated with PHQ-9 scores (r = 0.52, *p* < 0.001). Conclusions: Culture-confirmed infection, particularly candidemia, was associated with higher neonatal morbidity, markedly greater resource use, and substantial caregiver distress at discharge.

## 1. Introduction

Prematurity remains a leading driver of neonatal morbidity and mortality, largely because physiologic immaturity intersects with intensive care exposures that increase susceptibility to infection. In very preterm infants, suspected or culture-confirmed sepsis is not a discrete “intercurrent complication”; it often represents a turning point associated with escalating respiratory support, delayed enteral feeding progression, and prolonged hospitalization [1]. Contemporary multicenter data illustrate that late-onset sepsis remains a persistent burden even in modern NICU networks: in a large cohort of very preterm infants (2018–2020), late-onset sepsis was reported in 8.9% overall, with the highest incidence among those born at ≤23 weeks’ gestation (322.1 per 1000 infants) and decreasing steadily with advancing gestational age [2]. Earlier landmark network data similarly emphasized scale and impact: among very low-birth-weight infants surviving beyond 3 days, 21% experienced ≥1 episode of culture-proven late-onset sepsis, which was associated with longer hospitalization and higher mortality compared with their uninfected peers [3].

Neonatal sepsis in premature infants is clinically heterogeneous, and its epidemiology is shaped by developmental vulnerabilities and device-driven care. Pathogen patterns vary by gestational age, length of stay, and exposure to central lines, parenteral nutrition, and broad-spectrum antibiotics. Gram-positive organisms frequently reflect skin barrier immaturity and catheter-associated colonization, whereas Gram-negative pathogens may be linked to gastrointestinal translocation, dysbiosis, or environmental reservoirs in the NICU. Importantly, the clinical “signal” of infection can differ by organism and timing, and repeated infectious episodes may reflect cumulative iatrogenic risk rather than a single preventable event. Reviews of late-onset neonatal sepsis highlight the ongoing role of coagulase-negative staphylococci and the clinical importance of Gram-negative organisms in driving severe disease, while underscoring the need for prevention strategies that integrate infection control, antibiotic stewardship, and device minimization [4].

Beyond acute instability, sepsis may amplify inflammatory pathways that impair organ development during critical windows of maturation. Associations between late-onset sepsis and bronchopulmonary dysplasia (BPD) have been repeatedly observed, supporting the concept that systemic inflammation and prolonged ventilation interact to disrupt lung development [5]. Similarly, infection-related hemodynamic and inflammatory perturbations may increase vulnerability of the premature brain, and early-onset neonatal sepsis has been identified as a risk factor for peri-intraventricular hemorrhage in premature infants in observational analyses [6]. Retinal vascular development may also be affected: recent multicenter work in very preterm infants found that the number of neonatal sepsis episodes correlated with higher risk of retinopathy of prematurity (ROP), consistent with a dose–response relationship between systemic illness burden and retinal outcomes [7].

These short-term complications matter because they can translate into longer-term neurodevelopmental risk. Preterm infants who experience neonatal sepsis often face a higher likelihood of motor and cognitive challenges, and aggregated evidence suggests that infection exposure is associated with worse neurodevelopmental outcomes compared with their preterm peers without sepsis. A systematic review and meta-analysis focusing on very preterm infants reported adverse associations between neonatal sepsis and both short- and long-term neurodevelopmental outcomes, reinforcing the need to treat infection as a marker of both acute risk and longitudinal vulnerability [8]. Framing sepsis as a determinant of “trajectory,” rather than a transient diagnosis, is therefore clinically relevant for discharge planning, follow-up intensity, and family counseling.

Invasive fungal infection, most commonly invasive candidiasis (candidemia in the context of this study), occurs less frequently than bacterial sepsis but is often associated with disproportionately severe outcomes in extremely premature infants. Candidemia risk is closely linked to central venous catheters, parenteral nutrition, prolonged or repeated antibiotic exposure, and long-stay NICU phenotypes. In multicenter data on extremely low-birth-weight infants, neonatal candidiasis was associated with substantial mortality and concerning neurodevelopmental outcomes at 18–22 months, emphasizing the high clinical stakes of delayed recognition and treatment [9]. Reviews further note that invasive candidiasis typically presents with nonspecific signs of sepsis in the first weeks of life, and that standard blood cultures may miss a meaningful proportion of cases—highlighting the practical challenges of timely diagnosis in this population [10].

While neonatal outcomes remain central in NICU research, the NICU course can also exert a major psychosocial toll on caregivers. Systematic review evidence indicates that parents of infants admitted to NICU experience high prevalence of anxiety, depression, and stress symptoms, often intensified by recurrent clinical setbacks and uncertainty about prognosis [11]. Because caregiver mental health can shape engagement with care plans, feeding decisions, and readiness for discharge, measuring caregiver outcomes is increasingly aligned with family-centered care priorities. Generic quality-of-life tools such as the SF-36 can capture broad functional impact [12], while symptom-focused instruments such as the Hospital Anxiety and Depression Scale (HADS) [13], the Patient Health Questionnaire-9 (PHQ-9) [14], and the Generalized Anxiety Disorder-7 (GAD-7) survey [15] enable structured detection of clinically relevant distress that may merit referral or follow-up.

## 2. Materials and Methods

### 2.1. Study Design and Setting

This research project was structured as a single-center prospective observational cohort study conducted in the Neonatology/NICU environment affiliated with the “Victor Babeș” University of Medicine and Pharmacy Timișoara (UMFT) and its clinical partners in Timișoara, Romania. Enrollment occurred consecutively over a defined study window (March 2023–December 2025), with infants followed from NICU admission until discharge or in-hospital death. The goal was to evaluate infection-related differences in neonatal outcomes while simultaneously capturing caregiver psychosocial trajectories during hospitalization.

The protocol was designed to align with the ethical principles of the Declaration of Helsinki and standard NICU research governance. Institutional ethical approval was obtained, and written informed consent was collected from caregivers for questionnaire administration and chart-based data abstraction. Data were managed with de-identification procedures, limited access, and an audit trail. Because caregiver mental health instruments were included, the protocol did specify a referral pathway for clinically significant scores, ensuring that research screening does not replace clinical care.

### 2.2. Participants, Eligibility, and Group Definitions

The study included 87 premature infants admitted to the NICU. Eligibility criteria were designed to represent a clinically relevant high-risk population: premature birth (<34 weeks gestational age or NICU admission due to prematurity-related complications), NICU stay long enough to allow infection surveillance, and availability of caregiver participation for survey completion. Infants with major congenital anomalies incompatible with survival or with missing core outcome data were excluded to reduce heterogeneity unrelated to infection exposure.

Infants were categorized into three groups using culture-based definitions to strengthen interpretability. No proven infection required no positive blood culture during the NICU stay. Bacterial sepsis required at least one positive blood culture for bacterial pathogens consistent with clinical sepsis management. Candidemia was defined as a positive blood culture for Candida species accompanied by systemic antifungal treatment. For infants with multiple episodes, classification followed the most clinically significant infection category (candidemia superseding bacterial sepsis if both occurred). Timing metrics (day of first positive culture) were recorded relative to birth day to distinguish earlier- versus later-infection patterns.

### 2.3. Data Collection and Outcome Measures

Clinical data were abstracted from NICU records using a standardized case report form. Baseline infant variables included gestational age, birth weight, sex, multiple gestation, small-for-gestational-age status, and early illness severity indices. Perinatal exposures (antenatal steroids, delivery mode, prolonged rupture of membranes, chorioamnionitis) were included because they plausibly influence early instability and infection risk. Infection-related variables included day of first positive culture, number of infection episodes, organism category (Gram-positive/Gram-negative/polymicrobial for bacteria; Candida species for candidemia), and therapy markers (antibiotic days, antifungal days, prior broad-spectrum exposure).

Primary neonatal outcomes were selected to reflect both burden and severity: length of stay (LOS), days of mechanical ventilation, total respiratory support days, parenteral nutrition days, mortality prior to discharge, and major morbidities (moderate/severe BPD, NEC ≥ stage 2, severe IVH grade III–IV, severe ROP ≥ stage 3). A composite adverse outcome was defined as death or any major severe morbidity to provide a clinically meaningful summary endpoint. Outcomes were measured consistently across groups until discharge or death.

Caregiver-reported outcomes were captured using validated instruments: SF-36 (physical and mental component summaries), WHOQOL-BREF (psychological domain emphasized for this analysis), HADS, PHQ-9, GAD-7, and Body Image Scale (BIS). Surveys were administered at two standardized points: an early NICU timepoint (within the first 7–10 days) and a pre-discharge timepoint (during discharge planning, close to infant discharge or transition of care). One primary caregiver per infant was recorded to avoid duplicated family clustering in the main analyses.

### 2.4. Statistical Analysis

Analyses were conducted using standard biostatistical approaches appropriate for mixed outcome types. Continuous variables were summarized as mean ± SD (one decimal), and categorical variables as *n* (%). Group comparisons across the three infection categories used one-way ANOVA for continuous outcomes and χ^2^ tests for categorical outcomes; when comparing bacterial vs. candidemia for infection-specific metrics, Welch’s *t*-tests were used for continuous variables, and Fisher’s exact tests for binary variables due to small sample sizes in the candidemia group. All tests were two-tailed with a significance threshold of *p* < 0.05.

To examine predictors of high resource utilization, a binary endpoint of prolonged hospitalization (LOS ≥ 55 days) was modeled using logistic regression with infection group indicators and gestational age. Model results were reported as adjusted odds ratios (OR) with 95% confidence intervals. Associations between infant severity indicators and caregiver psychological outcomes were explored using Pearson correlation coefficients, focusing on clinically interpretable pairings (e.g., LOS with PHQ-9; CRIB-II with caregiver mental health).

## 3. Results

Across the three groups (no infection, bacterial sepsis, and candidemia (defined as blood-culture-confirmed Candida infection with systemic antifungal treatment)), most perinatal characteristics were broadly comparable, but illness severity and gestational maturity differed. Gestational age (GA) showed a statistically significant gradient, with the no-infection group having the highest GA (29.2 ± 1.7 weeks) compared with the bacterial sepsis (28.5 ± 1.5 weeks) and candidemia (28.0 ± 1.4 weeks; *p* = 0.034) groups. Birth weight was numerically lower in infected groups (1431.2 ± 392.9 g vs. 1321.8 ± 322.3 g vs. 1278.1 ± 327.5 g), but this difference was not significant (*p* = 0.278). Key obstetric exposures (cesarean delivery 59.1–66.7%, complete antenatal steroid course 66.7–74.2%, PROM 16.7–22.7%, and clinical chorioamnionitis 12.9–20.5%) did not differ meaningfully across groups (all *p* > 0.60), suggesting that infection status was not strongly driven by measured maternal/peripartum factors in this cohort. In contrast, baseline physiologic severity (SNAPPE-II) differed markedly, increasing from 22.9 ± 7.4 (no infection) to 26.8 ± 8.5 (bacterial sepsis) and 30.8 ± 7.6 (candidemia; *p* = 0.005), indicating that neonates who developed candidemia had the highest early severity of illness at baseline (Table 1).

Among infected neonates, candidemia was characterized by later onset and longer delays to appropriate therapy compared with bacterial sepsis. The day of life at first positive culture was substantially higher in candidemia (17.8 ± 4.8 days) than in bacterial sepsis (10.1 ± 3.9 days; *p* < 0.001), consistent with predominantly late-onset acquisition. Similarly, time to effective therapy was prolonged in candidemia (23.3 ± 9.5 h) versus bacterial sepsis (13.3 ± 5.3 h; *p* = 0.004), a clinically relevant gap that may reflect diagnostic complexity, slower recognition, or delayed initiation of targeted antifungal treatment. Early-onset infection (<72 h) was rare (1/31, 3.2%) and absent in candidemia (0/12), reinforcing the late-onset pattern. Bloodstream infection predominated in both groups (blood cultures: 80.6% bacterial vs. 66.7% candidemia), while mixed sites varied (blood + CSF 16.1% in bacterial sepsis; blood + urine 25.0% in candidemia), suggesting possible differences in dissemination and source. Exposure to broad-spectrum antibiotics ≥10 days before culture was very common and similar in both groups (90.3% vs. 91.7%), but documented fungal colonization was frequent before candidemia (75.0%), supporting colonization as an important pre-candidemia signal. Microbiology aligned with expected patterns: bacterial sepsis was mainly due to CoNS (32.3%), *Pseudomonas* spp. (22.6%), and *E. coli* (22.6%), whereas candidemia was entirely due to Candida, led by *C. albicans* (41.7%), followed by *C. tropicalis* (33.3%) and *C. parapsilosis* (25.0%), as described in Table 2.

Clinical outcomes were consistently worse in neonates with infection, with the largest relative increases seen in composite morbidity and selected complications. A composite adverse outcome occurred in 27.3% (12/44) of neonates without infection, but approximately doubled in bacterial sepsis (54.8%, 17/31) and was similarly high in candidemia (58.3%, 7/12), yielding a significant overall difference (*p* = 0.025). In-hospital mortality increased numerically with infection (11.4% vs. 16.1% vs. 25.0%), but this trend did not reach statistical significance (*p* = 0.489), likely reflecting limited power, especially in the candidemia subgroup. Morbidity patterns suggested infection-associated organ injury and complications: NEC (Bell stage ≥ II) was uncommon in the no-infection group (2.3%) but markedly higher with bacterial sepsis (19.4%) and candidemia (16.7%), reaching significance (*p* = 0.042). Severe IVH (grade III–IV) also increased stepwise (6.8% vs. 19.4% vs. 33.3%) and approached significance (*p* = 0.051), indicating a potential association that may become clearer in larger samples. ROP requiring treatment showed one of the strongest signals, rising from 4.5% (no infection) to 29.0% (bacterial sepsis) and 25.0% (candidemia; *p* = 0.012), consistent with greater systemic illness and prolonged intensive care exposure among infected neonates. Moderate-to-severe BPD was more frequent in infected groups (27.3% vs. 38.7% vs. 50.0%) but did not differ significantly (*p* = 0.281), as seen in Table 3.

Of note, while the overall three-group comparison was significant (*p* = 0.025), pairwise comparisons between no infection and candidemia, and between bacterial sepsis and candidemia, did not individually reach significance due to the limited sample size of the candidemia group (*n* = 12), which reduces statistical power despite numerically large differences in composite outcome rates.

Resource utilization increased in a stepwise fashion from no infection to bacterial sepsis to candidemia, indicating a progressively higher NICU burden associated with infectious complications. Length of stay rose from 39.7 ± 10.2 days (no infection) to 50.1 ± 11.9 days (bacterial sepsis) and 60.9 ± 13.1 days (candidemia), with strong evidence of group differences (*p* < 0.001). The same graded pattern was observed for invasive ventilation days (15.7 ± 7.6 vs. 23.3 ± 7.5 vs. 34.2 ± 10.4; *p* < 0.001) and total respiratory support days (28.2 ± 10.5 vs. 41.6 ± 13.5 vs. 46.2 ± 21.2; *p* < 0.001), consistent with greater pulmonary morbidity and prolonged dependence on support in infected neonates. Central-line days also increased substantially (16.2 ± 7.9 vs. 22.1 ± 9.0 vs. 26.6 ± 12.8; *p* < 0.001), aligning with higher exposure to invasive devices and potential infection risk. Parenteral nutrition days were numerically higher with infection (14.9 ± 7.6 vs. 18.5 ± 10.7 vs. 21.8 ± 9.4), but this did not reach statistical significance (*p* = 0.083). Antimicrobial exposure clearly escalated: systemic antibiotics increased from 11.6 ± 4.1 days to 18.2 ± 5.9 days and 23.6 ± 7.6 days (*p* < 0.001), while systemic antifungal exposure was minimal without infection (0.9 ± 0.9 days) and markedly higher in candidemia (15.6 ± 7.0 days; *p* < 0.001), reflecting treatment intensity and prolonged courses in fungal disease (Table 4).

Caregiver-reported health status and psychosocial outcomes at discharge were significantly worse when neonates experienced infection, with the most pronounced impairments in the candidemia group. On SF-36, physical health (PCS) decreased from 44.6 ± 7.2 (no infection) to 40.7 ± 6.4 (bacterial sepsis) and 37.9 ± 3.6 (candidemia; *p* = 0.003), while mental health (MCS) showed an even larger decline (44.7 ± 7.5 vs. 38.5 ± 6.0 vs. 36.7 ± 6.4; *p* < 0.001). WHOQOL-BREF domains similarly suggested reduced quality of life with infection: physical (56.1 ± 8.1 vs. 53.7 ± 6.5 vs. 50.1 ± 6.8; *p* = 0.044) and psychological (57.2 ± 7.3 vs. 52.2 ± 9.3 vs. 51.1 ± 7.6; *p* = 0.012) domains were significantly lower, and the environmental domain demonstrated a marked drop, especially in candidemia (65.8 ± 6.4 vs. 62.6 ± 6.5 vs. 56.5 ± 8.6; *p* < 0.001), potentially reflecting accumulated stressors and post-discharge logistical burden. Mental health symptom scale scores were consistently higher with infection: HADS-Anxiety scores increased from 9.8 ± 3.0 to 11.5 ± 3.5 and 13.9 ± 2.7 (*p* < 0.001), HADS-Depression scores from 8.8 ± 2.9 to 11.1 ± 2.9 and 12.5 ± 2.3 (*p* < 0.001), PHQ-9 scores from 9.4 ± 3.9 to 11.6 ± 3.3 and 15.5 ± 4.6 (*p* < 0.001), and GAD-7 scores from 8.8 ± 3.4 to 10.1 ± 3.0 and 14.1 ± 2.2 (*p* < 0.001). Body image concerns were also greater in candidemia (BIS 10.1 ± 2.6) versus no infection (6.6 ± 3.3; *p* = 0.002), as seen in Table 5.

To contextualize the clinical significance of these findings, we applied established instrument cut-off points for identifying caregivers with at least moderate symptom severity: PHQ-9 ≥ 10 (moderate depression), GAD-7 ≥ 10 (moderate anxiety), HADS-Anxiety ≥ 8, and HADS-Depression ≥ 8. The proportion of caregivers exceeding these thresholds increased progressively with infection severity: PHQ-9 ≥ 10 was met by 40.9% (no infection), 61.3% (bacterial sepsis), and 83.3% (candidemia; *p* = 0.021); GAD-7 ≥ 10 by 34.1%, 45.2%, and 91.7% (*p* = 0.002); HADS-Anxiety ≥ 8 by 56.8%, 74.2%, and 91.7% (*p* = 0.044); and HADS-Depression ≥ 8 by 52.3%, 77.4%, and 91.7% (*p* = 0.013). These data indicate that a substantial and increasing majority of caregivers in the candidemia group met or exceeded clinically actionable thresholds for depression and anxiety.

NICU burden, summarized as the first principal component (PC1), showed consistent associations with worse caregiver-reported mental health and greater symptom severity, and these relationships remained largely intact after adjustment for gestational age (GA). Higher PC1 correlated with lower SF-36 mental functioning (MCS), with a moderate negative correlation (r = −0.34, *p* = 0.001) that persisted after GA adjustment (partial r = −0.323, *p*_adj = 0.002). In contrast, higher PC1 was strongly associated with higher depression and anxiety symptom scores: PHQ-9 demonstrated the largest correlation (r = 0.52, *p* < 0.001; partial r = 0.502, *p*_adj < 0.001), and GAD-7 was similarly robust (r = 0.485, *p* < 0.001; partial r = 0.467, *p*_adj < 0.001). HADS subscales reinforced the pattern, with HADS-A (r = 0.428, *p* < 0.001; partial r = 0.406, *p*_adj < 0.001) and HADS-D (r = 0.475, *p* < 0.001; partial r = 0.452, *p*_adj < 0.001) indicating that greater NICU burden tracked with higher anxiety and depressive symptoms. Body image impairment also increased with PC1 (BIS r = 0.293, *p* = 0.006; partial r = 0.272, *p*_adj = 0.011), suggesting broader psychosocial impact beyond mood alone. Finally, PC1 was modestly associated with the composite adverse neonatal outcome (r = 0.259, *p* = 0.015), and the association attenuated but remained significant after GA adjustment (partial r = 0.218, *p*_adj = 0.043), indicating that NICU burden relates to both clinical morbidity and caregiver psychological outcomes, partly independent of prematurity severity (Table 6).

**Figure 1:**
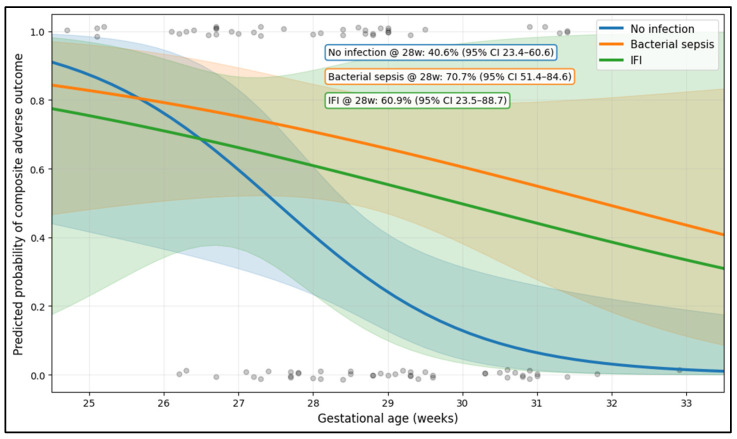
Adjusted risk curves for a composite adverse neonatal outcome across gestational age, stratified by infection group.

The adjusted logistic model shows a strong inverse association between gestational age and the probability of the composite adverse neonatal outcome, with consistently higher risk in infected groups compared with the no-infection group. At 28.0 weeks, the predicted probability was 40.6% (95% CI 23.4–60.6) for no infection, 70.7% (95% CI 51.4–84.6) for bacterial sepsis, and 60.9% (95% CI 23.5–88.7) for candidemia, highlighting a clear infection-related risk gradient at the same gestational age. By 30.0 weeks, predicted risk dropped substantially in the no-infection group to 12.8% (95% CI 4.5–31.3), while remaining higher in the bacterial sepsis (60.5%, 95% CI 38.2–79.1) and candidemia (49.7%, 95% CI 3.4–96.6) groups, indicating that infection status modifies overall risk beyond prematurity alone; the wider candidemia uncertainty reflects the smaller sample size (Figure 1).

**Figure 2:**
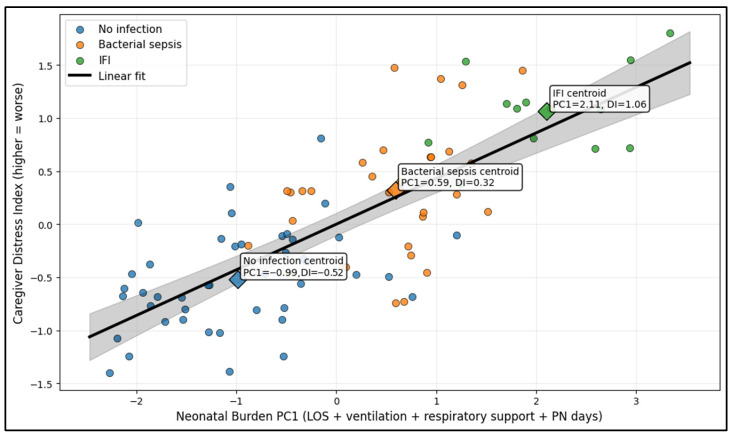
PCA-derived neonatal “burden” score vs. caregiver distress index at discharge, by infection group.

The multivariate analysis demonstrates that higher NICU “burden” (PC1 from LOS, ventilation days, respiratory support, and PN days) is strongly aligned with worse caregiver psychological distress at discharge. Neonatal Burden PC1 explained 46.2% of the variance in NICU utilization patterns, and it correlated strongly with the Caregiver Distress Index (r = 0.76, *p* < 0.001), meaning that infants with more intense/prolonged NICU courses tended to have caregivers reporting higher combined depression/anxiety/body-image burden (and lower mental health). Group centroids illustrate a stepwise shift: No infection clustered at lower burden and distress levels (PC1 = −0.99; DI = −0.52), bacterial sepsis at intermediate levels (PC1 = 0.59; DI = 0.32), and candidemia at the highest burden and distress level (PC1 = 2.11; DI = 1.06), suggesting that fungal infection was associated with the most resource-intensive courses and the highest caregiver strain in this cohort (Figure 2).

## 4. Discussion

### 4.1. Analysis of Findings

It should be noted that the overall rate of culture-confirmed infection in our cohort (49.4%) is considerably higher than the 8–21% late-onset sepsis rates reported in large Western multicenter networks. This elevated rate is consistent with historical surveillance data from our center, which serves as a tertiary referral NICU in Central–Eastern Europe, receiving a disproportionate share of extremely preterm and critically ill neonates with prolonged device exposure. Eligibility criteria that required sufficient NICU duration for infection surveillance may have further enriched the sample for higher-risk infants. Our findings should therefore be interpreted with caution when extrapolating to NICUs with different patient case-mixes, infection-control resources, or lower baseline infection rates.

In this cohort, the stepwise decrease in gestational maturity and the parallel increase in early physiologic severity (higher SNAPPE-II) among infected neonates likely reflect the well-described interaction between immaturity, invasive support needs, and infection vulnerability. Our finding that the no-infection group had a higher mean GA (29.2 weeks) than the bacterial sepsis (28.5 weeks) and candidemia (28.0 weeks) groups, alongside a marked rise in SNAPPE-II (22.9 → 26.8 → 30.8), supports the concept that infection is concentrated among the most fragile infants—those who enter the NICU already on a higher-risk trajectory. Large international comparisons in extremely preterm infants likewise show that late-onset sepsis remains common across high-income settings and is strongly patterned by gestational age, with earlier gestations experiencing the greatest burden [16]. Clinically, this matters for interpretation: although infection contributes to downstream morbidity, part of the observed risk gradient likely begins at baseline, where physiologic instability, prolonged vascular access, and early ventilation create both the exposure opportunities and the diminished physiologic reserve that make subsequent sepsis or candidemia more consequential. The implication is that prevention and early recognition efforts may yield the largest absolute benefit when targeted toward infants with the highest initial illness severity and the longest anticipated device exposure windows [16,17,18,19,20].

A key signal in our culture-based infection-type characterization was that candidemia occurred later (mean DOL 17.8 vs. 10.1) and was associated with substantially longer delays to effective therapy (23.3 vs. 13.3 h). This aligns with the clinical reality that invasive candidiasis often presents with nonspecific deterioration and may be harder to confirm quickly, especially when blood cultures are intermittently negative or slow to flag. While our dataset cannot determine causality, a delay of ~10 h in time to effective therapy is plausibly meaningful in extremely vulnerable infants, because fungal disease frequently overlaps with central-line dependence, parenteral nutrition, and heavy antecedent antibiotic exposure—exactly the phenotype we observed ≥10 days of broad-spectrum antibiotics in >90% of both infected groups, and documented fungal colonization in 75% of candidemia). Importantly, the Candida species distribution (*C. albicans*, *C. tropicalis*, *C. parapsilosis*) is consistent with NICU epidemiology, where both mucosal/skin colonization and line-associated biofilm biology play a role. From a prevention standpoint, the broader literature has evaluated fluconazole prophylaxis in VLBW/ELBW infants; meta-analytic evidence suggests that prophylaxis can reduce invasive candidiasis in high-risk populations, though policies must be individualized to local incidence and stewardship priorities [21,22,23,24].

Notably, the pathogen distribution in our cohort differs from patterns reported in large multicenter networks from high-income settings, where coagulase-negative staphylococci typically dominate late-onset sepsis, and invasive candidiasis rates generally remain below 10%. In our series, Gram-negative organisms (*Pseudomonas* spp. 22.6%, *E. coli* 22.6%, and *Klebsiella* spp. 9.7%) collectively accounted for more than half of bacterial isolates, and candidemia represented approximately 28% of all culture-confirmed infections. These elevated proportions likely reflect a combination of factors: our center serves as a tertiary referral NICU receiving critically ill, extremely preterm infants with prolonged device exposure and heavy antecedent antibiotic pressure—conditions known to select for resistant Gram-negative flora and to promote fungal colonization. Additionally, organism ecology and infection-control resources in Central and Eastern European NICUs may differ from those in large North American and Western European network studies. While this does not confirm deficiencies in infection control at our center, it signals that local prevention bundles—including enhanced hand hygiene compliance, catheter-care protocols, environmental decontamination, and targeted antifungal prophylaxis—deserve ongoing quality-improvement attention. The relatively small single-center denominator also amplifies proportions, and these contextual factors should be considered when comparing our pathogen frequencies with published network-level data.

The morbidity profile in our results is also concordant with the broader evidence linking infection to organ injury during critical developmental windows. The composite adverse outcome approximately doubled with infection (27.3% no infection vs. 54.8% bacterial sepsis and 58.3% candidemia), and we saw particularly strong signals for NEC and ROP requiring treatment. The NEC increase (2.3% vs. 19.4% vs. 16.7%) fits mechanistically with inflammation-driven intestinal vulnerability in preterm infants, and it is clinically notable that antibiotic exposure escalated sharply across groups. Landmark NICHD network data have shown that prolonged early empiric antibiotics are associated with higher NEC and death in ELBW infants, underscoring how antimicrobial pressure may amplify dysbiosis-mediated risk even when sepsis is not culture-proven [18]. Similarly, multicenter Canadian Neonatal Network analyses have associated broader antibiotic exposure with adverse neonatal outcomes among VLBW infants, reinforcing the importance of minimizing unnecessary duration and breadth [19]. For ROP, our marked increase in treatment-requiring disease with infection (4.5% vs. 29.0% vs. 25.0%) is consistent with contemporary etiologic frameworks proposing neonatal sepsis as a biologically plausible contributor via systemic inflammation and vascular dysregulation [21]. Finally, the near-significant stepwise increase in severe IVH (6.8% → 19.4% → 33.3%) highlights a pattern that has been linked in other cohorts to infection-related hemodynamic instability and inflammatory injury, and multiple studies report worse neurodevelopment after neonatal sepsis in extremely preterm populations at 2 years of age [22,23].

Resource utilization in our cohort rose dramatically from no infection to bacterial sepsis to candidemia—length of stay (+~21 days from no infection to candidemia), invasive ventilation (15.7 → 34.2 days), respiratory support, and central-line days all increased, with strong statistical signals (*p* < 0.001 for several domains). This “dose” of intensive care exposure likely represents both cause and consequence: prolonged respiratory support and vascular access increase infection risk, while infection episodes can delay feeding advancement, prolong ventilation, and extend catheter dependence. Our observed pattern also aligns with evidence connecting late-onset sepsis phenotypes to later pulmonary morbidity; for example, clinical studies have reported associations between late-onset sepsis characteristics and subsequent bronchopulmonary dysplasia in preterm infants, supporting the idea that systemic inflammatory hits compound ventilator-related lung injury [17]. From a practice standpoint, these data support bundled approaches that treat infection prevention and “device minimization” as inseparable goals—line necessity audits, early transition to enteral nutrition when feasible, and stewardship strategies that reduce collateral microbiome injury without compromising timely coverage for true sepsis [24,25].

A distinctive contribution of this study is the parallel measurement of caregiver outcomes and the quantified linkage between NICU burden and caregiver distress. We found clinically meaningful declines in caregiver physical and especially mental health (SF-36 MCS 44.7 → 38.5 → 36.7) and substantial increases in symptom scales (e.g., PHQ-9 9.4 → 11.6 → 15.5; GAD-7 8.8 → 10.1 → 14.1) with the most pronounced impairments in the candidemia group. These findings are consistent with global meta-analytic evidence showing high parental stress during NICU hospitalization, with infant medical complexity and prolonged admission intensifying stress exposure [26]. The Parent Stress Scale–NICU framework and related stress constructs have been used to characterize how “sights and sounds,” altered the parental role, and infant appearance/behavior contribute to distress [27]. Importantly, the literature on intervention suggests that structured family-integrated care models can improve both parent and infant outcomes in multicenter trials, supporting actionable pathways beyond screening alone [25]. In light of our PC1 correlations (burden correlating strongly with depression/anxiety and lower mental functioning), a practical implication is to couple risk-stratified psychosocial screening at discharge with targeted support for families whose infants experienced infection and prolonged intensive care courses—especially where candidemia signals a “highest-burden” phenotype.

These findings support treating culture-confirmed infection in preterm infants as a “trajectory-changing” event that should trigger both intensified neonatal risk management and structured family support. Clinically, the later presentation and longer time to effective therapy in candidemia (17.8 days and 23.3 h) argues for NICU pathways that shorten antifungal decision latency in high-risk infants (those with prolonged central-line exposure, heavy prior use of broad-spectrum antibiotics, and documented fungal colonization). Because infection groups had substantially higher composite adverse outcomes and longer LOS/ventilation, discharge planning should anticipate greater follow-up needs (ROP surveillance, neurodevelopmental monitoring, respiratory care). In parallel, the clear gradient in caregiver mental health (MCS decline; PHQ-9 increase to 15.5 in candidemia) supports routine caregiver screening (PHQ-9/GAD-7 or HADS) near discharge with a predefined referral and follow-up workflow, integrating psychology/social work into family-centered NICU care. Nevertheless, these findings should be interpreted within the clinical context, as observed associations may be influenced by factors such as individual patient comorbidities, baseline illness severity, variability in clinical decision-making regarding antimicrobial initiation and duration, unmeasured socioeconomic determinants, and differences in parental coping resources that were not fully captured by the instruments employed [28,29,30,31,32,33,34].

### 4.2. Study Limitations

This was a single-center cohort, which may limit external generalizability because organism ecology, antifungal practices, and family-support resources vary across NICUs. The candidemia group was small, reducing power for some clinical endpoints (mortality) and widening uncertainty around estimates for the rarest outcomes. Although analyses incorporated standardized group definitions and gestational age adjustment in key models, residual confounding is likely (unmeasured illness complexity, socioeconomic factors, baseline caregiver mental health history). Infection classification relied on culture-confirmed (proven) sepsis as the reference standard; consequently, infants with clinical signs of infection but negative blood cultures were classified in the no-infection group, which may underestimate the true burden of sepsis in this cohort rather than misclassify individual episodes. Finally, caregiver outcomes were captured at standardized hospitalization timepoints but do not establish long-term trajectories beyond discharge.

## 5. Conclusions

In this prospective NICU cohort, both bacterial sepsis and candidemia were associated with markedly higher neonatal morbidity and resource utilization compared with no proven infection, with candidemia showing later onset and longer delays to effective therapy. Importantly, worse neonatal courses were accompanied by deterioration in caregiver-reported mental health that crossed established clinical thresholds for moderate depression and anxiety and higher depression/anxiety symptom scores at discharge, with NICU burden correlating strongly with caregiver distress. These data reinforce a dual-target approach: optimize infection prevention and rapid, appropriate therapy, especially for candidemia-risk phenotypes, while embedding routine caregiver mental health screening and support into NICU discharge planning.

## Figures and Tables

**Table 1:** Baseline neonatal and perinatal characteristics by infection group.

Variable	No Infection (*n* = 44)	Bacterial Sepsis (*n* = 31)	Candidemia (*n* = 12)	*p* (3-Group)
GA, weeks	29.2 ± 1.7	28.5 ± 1.5	28.0 ± 1.4	0.034
Birth weight, g	1431.2 ± 392.9	1321.8 ± 322.3	1278.1 ± 327.5	0.278
Male sex	21 (47.7%)	18 (58.1%)	5 (41.7%)	0.544
Cesarean delivery	26 (59.1%)	19 (61.3%)	8 (66.7%)	0.891
Antenatal steroids (complete course)	31 (70.5%)	23 (74.2%)	8 (66.7%)	0.875
Multiple gestation	5 (11.4%)	3 (9.7%)	2 (16.7%)	0.812
PROM	10 (22.7%)	7 (22.6%)	2 (16.7%)	0.897
Clinical chorioamnionitis	9 (20.5%)	4 (12.9%)	2 (16.7%)	0.694
SNAPPE-II	22.9 ± 7.4	26.8 ± 8.5	30.8 ± 7.6	0.005

Data are presented as mean ± SD or *n* (%). Abbreviations: GA, gestational age; candidemia, blood-culture-confirmed Candida infection; PROM, premature rupture of membranes; SNAPPE-II, Score for Neonatal Acute Physiology with Perinatal Extension II.

**Table 2:** Infection timing, source, and microbiology among infected neonates.

Variable	Bacterial Sepsis (*n* = 31)	Candidemia (*n* = 12)	*p* (2-Group)
Day of life at first positive culture	10.1 ± 3.9	17.8 ± 4.8	<0.001
Time to effective therapy, hours	13.3 ± 5.3	23.3 ± 9.5	0.004
Early-onset infection (<72 h)	1 (3.2%)	0 (0.0%)	1
Culture-positive site: blood	25 (80.6%)	8 (66.7%)	0.082
Culture-positive site: Blood + CSF	5 (16.1%)	1 (8.3%)	
Culture-positive site: blood + urine	1 (3.2%)	3 (25.0%)	
Broad-spectrum antibiotics ≥ 10 days before culture	28 (90.3%)	11 (91.7%)	1
Documented fungal colonization before candidemia	—	9 (75.0%)	
Pathogen (bacterial): CoNS	10 (32.3%)	—	
Pathogen (bacterial): *Pseudomonas* spp.	7 (22.6%)	—	
Pathogen (bacterial): *E. coli*	7 (22.6%)	—	
Pathogen (bacterial): *Enterococcus* spp.	3 (9.7%)	—	
Pathogen (bacterial): *Klebsiella* spp.	3 (9.7%)	—	
Pathogen (bacterial): *S. aureus*	1 (3.2%)	—	
Pathogen (fungal): *Candida albicans*	—	5 (41.7%)	
Pathogen (fungal): *Candida tropicalis*	—	4 (33.3%)	
Pathogen (fungal): *Candida parapsilosis*	—	3 (25.0%)	

Data are presented as mean ± SD or *n* (%). Abbreviations: CSF, cerebrospinal fluid; CoNS, coagulase-negative staphylococci; blood-culture-confirmed Candida infection.

**Table 3:** Major neonatal clinical outcomes by infection group.

Outcome	No Infection (*n* = 44)	Bacterial Sepsis (*n* = 31)	Candidemia (*n* = 12)	*p* (3-Group)
Composite adverse outcome *	12 (27.3%)	17 (54.8%)	7 (58.3%)	0.025
In-hospital mortality	5 (11.4%)	5 (16.1%)	3 (25.0%)	0.489
Moderate-to-severe BPD (grade ≥ 2)	12 (27.3%)	12 (38.7%)	6 (50.0%)	0.281
NEC (Bell stage ≥ II)	1 (2.3%)	6 (19.4%)	2 (16.7%)	0.042
Severe IVH (grade III–IV)	3 (6.8%)	6 (19.4%)	4 (33.3%)	0.051
ROP requiring treatment	2 (4.5%)	9 (29.0%)	3 (25.0%)	0.012

Data are presented as *n* (%). * Composite adverse outcome defined as any of in-hospital mortality, moderate-to-severe BPD (grade ≥ 2), NEC (Bell stage ≥ II), severe IVH (grade III–IV), or ROP requiring treatment. Abbreviations: BPD, bronchopulmonary dysplasia; candidemia, blood-culture-confirmed Candida infection; IVH, intraventricular hemorrhage; NEC, necrotizing enterocolitis; ROP, retinopathy of prematurity.

**Table 4:** NICU resource utilization and antimicrobial exposure by infection group.

Variable	No Infection (*n* = 44)	Bacterial Sepsis (*n* = 31)	Candidemia (*n* = 12)	*p* (Kruskal–Wallis)
Length of stay, days	39.7 ± 10.2	50.1 ± 11.9	60.9 ± 13.1	<0.001
Invasive ventilation, days	15.7 ± 7.6	23.3 ± 7.5	34.2 ± 10.4	<0.001
Respiratory support, days	28.2 ± 10.5	41.6 ± 13.5	46.2 ± 21.2	<0.001
Parenteral nutrition, days	14.9 ± 7.6	18.5 ± 10.7	21.8 ± 9.4	0.083
Central-line days	16.2 ± 7.9	22.1 ± 9.0	26.6 ± 12.8	<0.001
Systemic antibiotic exposure, days	11.6 ± 4.1	18.2 ± 5.9	23.6 ± 7.6	<0.001
Systemic antifungal exposure, days	0.9 ± 0.9	2.5 ± 2.8	15.6 ± 7.0	<0.001

Data are presented as mean ± SD. *p*-values are derived from Kruskal–Wallis testing. Abbreviations: NICU, neonatal intensive care unit; candidemia, blood-culture-confirmed Candida infection. Antimicrobial exposure is defined as the total number of calendar days on which systemic antibiotic or antifungal agents were administered, and does not represent defined daily doses (DDD) or pharmacoepidemiological consumption metrics.

**Table 5:** Caregiver-reported outcomes at discharge by neonatal infection group.

Measure (Caregiver at Discharge)	No Infection (*n* = 44)	Bacterial Sepsis (*n* = 31)	Candidemia (*n* = 12)	*p* (ANOVA)
SF-36 Physical Component Summary (PCS)	44.6 ± 7.2	40.7 ± 6.4	37.9 ± 3.6	0.003
SF-36 Mental Component Summary (MCS)	44.7 ± 7.5	38.5 ± 6.0	36.7 ± 6.4	<0.001
WHOQOL-BREF Physical	56.1 ± 8.1	53.7 ± 6.5	50.1 ± 6.8	0.044
WHOQOL-BREF Psychological	57.2 ± 7.3	52.2 ± 9.3	51.1 ± 7.6	0.012
WHOQOL-BREF Social	62.7 ± 8.0	60.1 ± 8.8	56.8 ± 11.3	0.105
WHOQOL-BREF Environmental	65.8 ± 6.4	62.6 ± 6.5	56.5 ± 8.6	<0.001
HADS-A	9.8 ± 3.0	11.5 ± 3.5	13.9 ± 2.7	<0.001
HADS-D	8.8 ± 2.9	11.1 ± 2.9	12.5 ± 2.3	<0.001
PHQ-9	9.4 ± 3.9	11.6 ± 3.3	15.5 ± 4.6	<0.001
GAD-7	8.8 ± 3.4	10.1 ± 3.0	14.1 ± 2.2	<0.001
Body Image Scale (BIS)	6.6 ± 3.3	7.7 ± 2.4	10.1 ± 2.6	0.002

Data are presented as mean ± SD. Abbreviations: ANOVA, analysis of variance; BIS, Body Image Scale; GAD-7, Generalized Anxiety Disorder-7; HADS-A, Hospital Anxiety and Depression Scale—Anxiety; HADS-D, Hospital Anxiety and Depression Scale—Depression; MCS, Mental Component Summary; PCS, Physical Component Summary; PHQ-9, Patient Health Questionnaire-9; SF-36, 36-Item Short Form Survey; WHOQOL-BREF, World Health Organization Quality of Life—BREF.

**Table 6:** Correlations between NICU Burden PC1 and outcomes.

Outcome/Scale	r (PC1)	*p*	Partial r (adj GA)	*p*_adj
SF-36 MCS	−0.34	0.001	−0.323	0.002
PHQ-9	0.52	<0.001	0.502	<0.001
GAD-7	0.485	<0.001	0.467	<0.001
HADS-A	0.428	<0.001	0.406	<0.001
HADS-D	0.475	<0.001	0.452	<0.001
BIS	0.293	0.006	0.272	0.011
Composite adverse outcome	0.259	0.015	0.218	0.043

GA, gestational age; GAD-7, Generalized Anxiety Disorder-7; HADS-A, Hospital Anxiety and Depression Scale—Anxiety; HADS-D, Hospital Anxiety and Depression Scale—Depression; MCS, Mental Component Summary (SF-36); PC1, first principal component (NICU burden); PHQ-9, Patient Health Questionnaire-9; r, correlation coefficient; *p*_adj, *p*-value after adjustment for GA.

## Data Availability

The data presented in this study are available on request from the corresponding author.
